# A DNA synthesis inhibitor is protective against proteotoxic stressors via modulation of fertility pathways in *Caenorhabditis elegans*

**DOI:** 10.18632/aging.100605

**Published:** 2013-10-10

**Authors:** Suzanne Angeli, Ida Klang, Renuka Sivapatham, Karla Mark, David Zucker, Dipa Bhaumik, Gordon J. Lithgow, Julie K. Andersen

**Affiliations:** Buck Institute for Research on Aging, 8001 Redwood Blvd. Novato, CA 94945 USA

**Keywords:** FUdR, germline, protein misfolding, reproduction

## Abstract

Loss of germline precursor cells in *C. elegans* has previously been shown to improve protein homeostasis and extend lifespan, possibly due to reallocation of resources to somatic cells. In contrast, mutants that are sterile simply due to loss of sperm or oocyte production have a normal lifespan, often leading to the conclusion that loss of reproduction per se may have minor effects on *C. elegans*. We have found that inhibiting reproduction in *C. elegans* via the DNA synthesis inhibitor 5-fluoro-2-deoxyuridine (FUdR) improves protein homeostasis, stress resistance, and healthspan in wild-type animals. We find that FUdR is dependent on oogenesis and oocytic maturation. The effects of FUdR are dependent on FEM pathways, which regulate initiation of spermatogenesis. Loss of FEM expression leads to feminized animals that maintain arrested oocytes and are refractory to the effects of FUdR. FUdR-dependence is restored by spermatogenic signals, which trigger oocytic maturation and ovulation. Further, loss of FEM-3, a novel protein required for spermatogenesis, is sufficient to improve aspects of proteostasis. These effects are independent of previously described germline signals, including the DAF-16/FOXO, DAF-12/VDR, and HSF-1 pathways. These findings suggest that genetic or chemical inhibition of oocyte production can improve protein homeostasis in *C. elegans*.

## INTRODUCTION

The ‘disposable soma’ theory postulates that reproduction requires a large amount of energy expenditure, which is likely to divert or reallocate resources from somatic cells and accelerate organismal aging [1]. In support of this theory, loss of germline stem cells in the hermaphroditic nematode *C. elegans* increases longevity and improves stress resistance and protein homeostasis [2-5]. Loss of germline stem cells modulates expression of the FOXO-like forkhead transcription factor, DAF-16, which directly impacts longevity and stress resistance [2-5]. In contrast, animals that are sterile due to loss of oocyte/sperm production or administration of the DNA synthesis inhibitor, 5-fluoro-2-deoxyuridine (FUdR), have lifespans similar to wild-type animals, suggesting that loss of fertility per se may have negligible effects on the organism [3, 6]. However, mounting evidence suggests that inhibition of fertility downstream of germline signaling can also have significant biological consequences on an organism. For example, sterile mutants or animals treated with FUdR both exhibit enhanced resistance to bacterial pathogens and increased survival under anoxic conditions [7, 8]. Administration of FUdR significantly extends the lifespan of animals with *tub-1* mutations, which exhibit fat accumulation, and *gas-1* mutations, which disrupt mitochondrial complex I function [6, 9]. FUdR also significantly alters the metabolomic profiles of both wild-type animals and long-lived *daf-2* mutants [10]. Thus, inhibition of reproduction can increase some forms of stress resistance and synergize with genetic alterations to extend lifespan.

Aged animals are characterized by the progressive decline of motor ability and the accumulation of fluorescent age-pigments and insoluble proteins [11-14]. Growing evidence suggests that improving protein homeostasis may not only be beneficial for diseases of protein misfolding, such as Alzheimer's disease, but may also regulate longevity and healthspan in wild-type animals [13-15]. Thus, pharmacological manipulations that improve protein homeostasis are of therapeutic interest. Given that recent studies show that animals that lack germ cells also exhibit improved protein homeostasis [4, 5], compounds that alter reproduction may have similar effects. In this study, we report that inhibition of reproduction via the small molecule DNA synthesis inhibitor FUdR can significantly alter aging phenotypes by improving protein homeostasis, stress resistance, and healthspan.

## RESULTS

### Fluorouridine analogs are protective against proteotoxic stress

**Figure 1:**
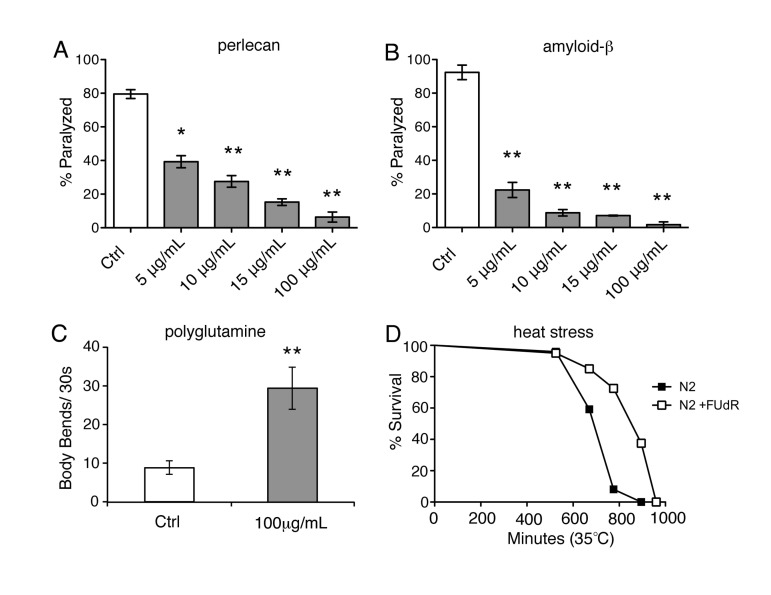
FUdR is protective under various conditions of proteotoxic stress **A**, Temperature-sensitive strain, HE250 [*unc-52(e669su250)II*], which expresses the misfolded basement membrane protein, perlecan, was scored for paralysis 24 hours after shifting to the restrictive temperature of 25°C (day 1 of adulthood) in the absence or presence of increasing concentrations of FUdR. FUdR-treatment rescues paralysis at all concentrations. (*=p<0.05, **=p<0.01, Student's t-test) **B**, Temperature-sensitive strain, CL4176 [*smg-1 (cc546ts); dvIs27(myo-3::Aβ3−42 let 39UTR(pAF29))*], which expresses amyloid-β_3-42_ peptide, was scored for paralysis on day 5 of adulthood in the absence or presence of increasing concentrations of FUdR. FUdR-treatment rescues paralysis at all concentrations. (**=p<0.01, Student's t-test) **C**, Temperature-sensitive strain, AM141 [*rmIs133(P(unc-54) Q40::YFP)*], which expresses a polyglutamine stretch of 40 repeats, was transiently exposed to FUdR during spermatogenesis and scored for motility on day 5 of adulthood. FUdR-treatment (100 μg/ml) significantly improves motility as measured by number of body bends. (**=p<0.001, Student's t-test) **D**, FUdR-treatment (100 μg/ml) significantly extends the survival of wild-type N2 animals at 35°C. (p=<0.001, Log rank Mantel-Cox).

We previously screened 640-compound natural-product library (NPL) for small molecules that improved phenotypes in models of protein misfolding and identified 5-fluorouridine (FU) as a hit (data not shown). Given the structural similarity between 5'FU and 5-fluoro 2-deoxyuridine (FUdR), we sought to recapitulate our findings with FUdR. We examined a temperature-sensitive strain (HE250 [*unc-52(e669su250)II*]) that exhibits misfolding of the endogenous basement membrane protein, perlecan, resulting in destabilization of muscle tissue with ensuing paralysis [16]. We shifted young adults to the restrictive temperature of 25°C and observed that approximately 80% of animals became paralyzed within 24 hours (Fig. 1A). We administered FUdR to young adult animals at a range of concentrations that are typically used to induce sterilization (5-100 μg/ml) and observed that FUdR-treatment significantly reduced the number of animals that became paralyzed in a dose-dependent manner (Fig. 1A). To determine if FUdR conferred protection in age-dependent models of protein misfolding, we examined a temperature-sensitive strain (CL4176 [*smg-1(cc546ts);dvIs27(myo-3::Aβ3-42 let 39UTR(pAF29))*]) in which the toxic peptide associated with Alzheimer's disease, Aβ, is expressed in muscle and leads to eventual paralysis [17]. By day 5 of adulthood, over 80% of animals became paralyzed at the restrictive temperature of 25°C (Fig. 1B). However, animals treated with FUdR throughout adulthood showed a dramatic dose-dependent decrease in paralysis (Fig. 1B). Thus, administration of FUdR effectively inhibits pathologies associated with protein misfolding in *C. elegans*.

Next, we sought to determine if transiently administering FUdR during the period of spermatogenesis was sufficient to inhibit paralysis in animals expressing Aβ(CL4176). Spermatogenesis in *C. elegans* occurs at the 4^th^ larval stage (L4), just before adulthood. Exposing animals to FUdR during this period inhibits sperm production and subsequently fertilized eggs are not produced. CL4176 animals were transiently exposed to FUdR for 24 hours during spermatogenesis and subsequently transferred to regular NGM plates for the remainder of the time. Transient exposure to FUdR (100 μg/ml) was sufficient to protect animals from age-dependent paralysis (Suppl. Fig. 1), similar to animals that were continuously exposed to FUdR (Fig. 1B). To test the generality of this observation, we also examined a temperature-sensitive strain (AM141 [*rmIs133(P(unc-54) Q40::YFP)*]) in which an extended polyglutamine stretch, associated with the onset of Huntington's disease, is expressed in the muscle [18]. As worms age at 25°C, large polyglutamine inclusions form, which greatly inhibits their motility [18]. Transient exposure to FUdR was sufficient to significantly improve the motility of aged animals expressing polyglutamine inclusions (Fig. 1C). Thus, inhibition of reproduction due to transient exposure to FUdR during spermatogenesis is sufficient to suppress pathologies associates with protein misfolding.

### FUdR confers thermotolerance and increases hsp16.2 expression

**Figure 2:**
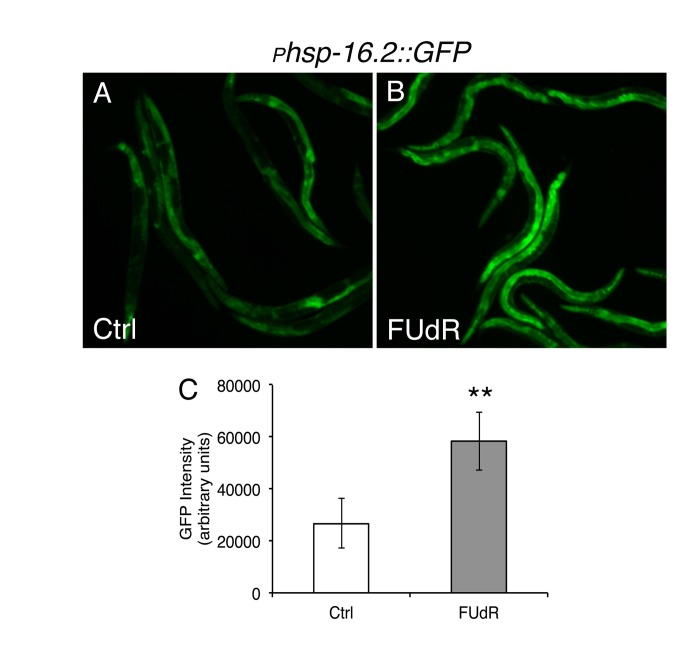
FUdR upregulates transcriptional expression of the heat shock regulated chaperone *hsp16.2* after heat stress **A**, **B**, Fluorescent photomicrographs of animals expressing the transcriptional reporter, *_p_hsp16.2::GFP*, after heat stress in the absence or presence of FUdR (100 μg/ml). Worms were heat stressed for 1 hour at 35°C and GFP expression was analyzed approximately 5 hours later. **C**, Quantification of GFP expression from A, B. FUdR-treatment significantly increases GFP levels compared to untreated control animals. (**=p<0.0001, Student's t-test)

Given that FUdR appears to protect against protein misfolding, we sought to determine if FUdR is also protective under other conditions of proteotoxic stress, such as heat stress. We continuously exposed animals to lethal heat stress (35°C) in the absence or presence of FUdR and assessed survival. FUdR-treatment significantly increased the survival of animals compared to untreated wild-type animals at 35°C (Fig. 1D, Suppl. Table 1). Thermotolerance can be conferred by overexpression of members of the heat shock chaperone family, which includes HSP-16 [19]. To determine if FUdR acts to modulate chaperone activity, we examined levels of the expression of heat shock chaperone *hsp-16.2* in FUdR-treated animals after heat stress. We utilized the transcriptional reporter strain, *_p_hsp-16.2::GFP*, in which GFP is under the transcriptional control of the *hsp-16.2* promoter [20]. Young adult animals were heat stressed at 35°C for 1 hour in the absence or presence of FUdR (100 μg/ml). Within 4-5 hours, animals exposed to FUdR showed significantly higher levels of GFP compared to controls (Fig. 2). We observed this upregulation at concentrations as low as 15 μg/ml of FUdR (data not shown). Thus, FUdR may confer thermotolerance in part by upregulating the heat stress response.

### FUdR confers thermotolerance via germline signals independent of daf-12, daf-16, and hsf-1

**Figure 3:**
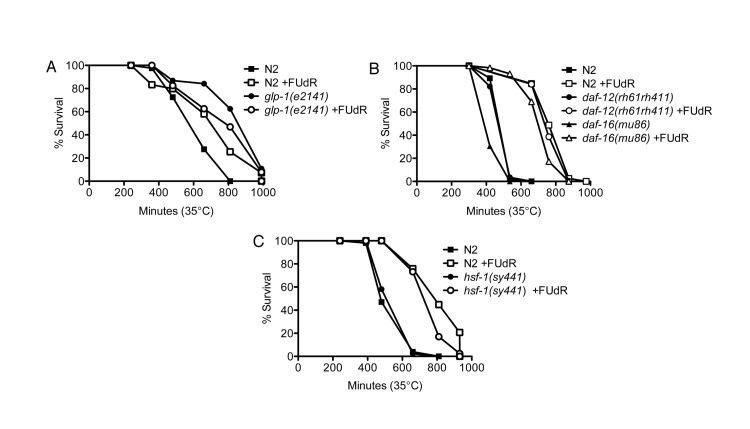
FUdR confers thermotolerance via germline signals that are independent of *daf-12, daf-16*, and *hsf-1* **A**, FUdR-treatment significantly extends wild-type N2 survival at 35°C while it is slightly toxic to germline-deficient *glp-1(e2141)* mutants. Animals were reared at the restrictive temperature of 25°C and shifted to 35°C as young adults. **B**, FUdR-treatment significantly extends *daf-12(rh61rh411)* and *daf-16(mu86)* survival at 35°C to a similar extent as wild-type N2 animals. Animals were reared at 20°C and shifted to 35°C as young adults. **C**, FUdR-treatment significantly extends *hsf-1(sy441)* survival at 35°C to a similar extent as wild-type N2 animals. Animals were reared at 20°C and shifted to 35°C as young adults. In all experiments, FUdR was used at 100 μg/ml.

Next, we tested which genetic pathway(s) FUdR requires to confer stress resistance. First, we tested whether FUdR confers thermotolerance by inhibiting cycling of germline stem cells, which has previously been shown to confer protection against heat stress [3]. We tested animals with a mutation in *glp-1*, a member of the Notch family of receptors [21], which, when reared at the restrictive temperature of 25°C, do not form a germline [22]. While FUdR-treatment significantly increased the survival of wild-type animals reared at 25°C, FUdR conferred no additional thermotolerance to the long-lived *glp-1(e2141)* mutants and actually made the worms slightly sensitive (Fig. 3A, Suppl. Table 1), indicating that the effects of FUdR result from a germline signal. Previous reports have shown that DAF-16, a FOXO transcription factor, and DAF-12, a steroid hormone receptor, are required for lifespan extension in germline-ablated or *glp-1(e2141)* mutant animals [2, 3]. Furthermore, *daf-16* is required for thermal resistance in long-lived animals [23]. Thus, we tested whether *daf-16* or *daf-12* were required for FUdR-induced thermotolerance. Interestingly, we found that FUdR-treatment conferred thermotolerance in animals carrying the *daf-16(mu86)* or *daf-12(rh61rh411)* null mutations to a similar extent as wild-type animals (Fig. 3B, Suppl. Table 1), indicating that FUdR's action does not arise from modulation of these previously characterized germline signals. Other pathways that have been reported to regulate germline signaling include fatty acid metabolism via the nuclear hormone receptor, *nhr-80*[24]. We tested the effects of FUdR on *nhr-80(tm1011)* mutants and found that FUdR significantly extended the survival of mutants to a similar extent as wild-type animals at 35°C (Suppl. Fig. 2), indicating that germline signaling via fatty acid metabolism was not required for FUdR-induced thermotolerance. Thus, FUdR is not acting via these previously described germline signaling pathways to modulate thermotolerance.

Given that FUdR upregulates *hsp16.2* expression, we sought to determine if the effects of FUdR required *hsf-1*, the gene encoding the heat shock factor ortholog. Surprisingly, we found that FUdR-treatment conferred thermotolerance in *hsf-1(sy441)* mutants to a similar extent as wild-type animals (Fig. 3C, Suppl. Table1). *hsf-1(sy441)* mutants do not contain a null mutation for *hsf-1* as it is thought to be embryonic lethal, but animals do exhibit severely dampened activation of HSP-16.2 after heat stress [23, 25]. Thus, it is possible that other functions of *hsf-1* may remain active to promote thermotolerance in the presence of FUdR. Alternatively, *hsp16.2* may be activated by pathways that are independent of *hsf-1*.

### FUdR confers thermotolerance via inhibition of oogenesis and oocytic maturation

**Figure 4:**
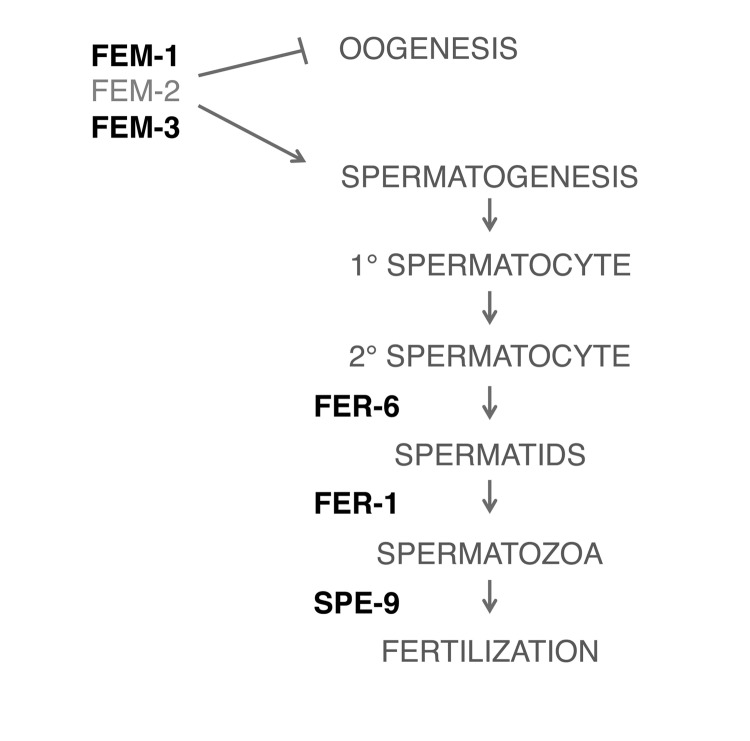
Simplified schematic of pathways involved in spermatogenesis Bolded pathways were tested with genetic mutants in the presence of FUdR (100 μg/ml) in thermotolerance assays.

**Figure 5:**
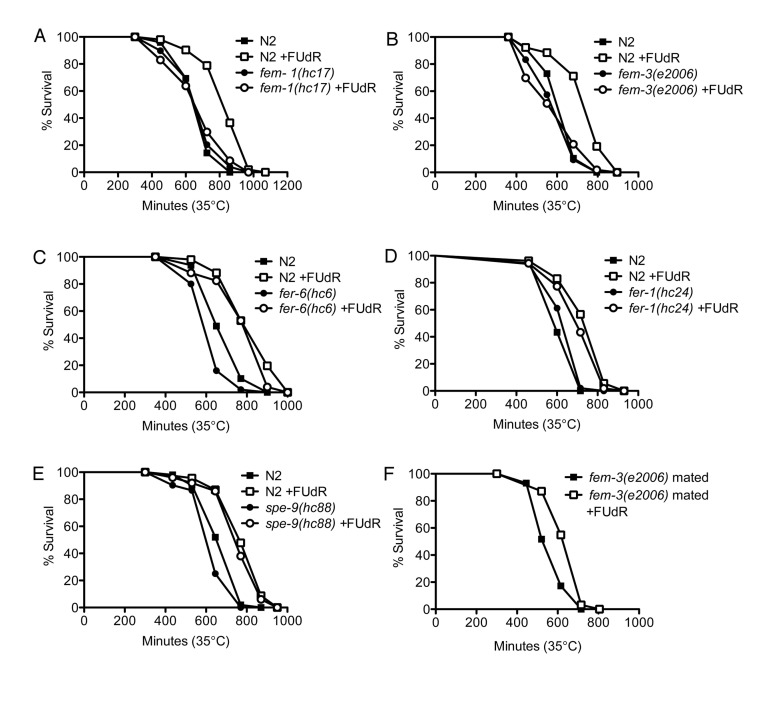
FUdR is dependent on oocytic maturation to confer stress resistance **A,B**, FUdR-treatment has no significant effect on *fem-1(hc17)* or *fem-3(e2006)* mutants while it significantly extends wild-type N2 survival at 35°C. **C-E**, FUdR-treatment significantly extends *fer-6(hc6), fer-1(hc24)*, and *spe-9(hc88)* survival at 35°C to a similar extent as wild-type N2 animals. **F**, FUdR-treatment significantly extends the survival of mated *fem-3(e2006)* mutants at 35°C. All temperature-sensitive mutants were reared at 25°C and shifted to 35°C as young adults. In all experiments, FUdR was used at 100 μg/ml.

We next sought to determine if FUdR may modulate other fertility signals to confer thermotolerance. We tested various fertility mutants to determine where FUdR may be acting (Fig. 4). First, we tested whether the sex-determining genes, *fem-1* and *fem-3*, which regulate male sex determination and initiation of spermatogenesis [26, 27], were required for the effects of FUdR. *fem-1(hc17)* and *fem-3(e2006)* mutants are phenotypically female, meaning that they form a female germline that lacks sperm or sperm precursors. Consequently, oocytes arrest in diakinesis of meiotic prophase and animals lay few to no unfertilized oocytes [28]. We found that FUdR did not confer thermotolerance to *fem-1(hc17)* or *fem-3(e2006)* mutants (Figs. 5A,B, Suppl. Table 1), indicating that the FEM signaling pathway is essential for FUdR-induced thermotolerance. To determine if all fertility mutants were recalcitrant to FUdR, we tested mutants with defects in spermatogenesis. *fer-6(hc6)* and *fer-1(hc24*) mutants initiate spermatogenesis, but are defective in the formation of spermatids or spermatozoa, respectively (Fig. 4) [29]. *spe-9(hc88)* mutants form mature spermatozoa, but are defective in binding to oocytes and completing fertilization (Fig. 4) [30]. The presence of spermatids or defective spermatozoa has previously been shown to be sufficient to trigger oocytic maturation and ovulation [28]. Thus, *fer-6(hc6)*, *fer-1(hc24)*), and *spe-9(hc88)* mutants animals can lay unfertilized oocytes at rates similar to wild-type animals [30]. Interestingly, we found that FUdR was able to confer thermotolerance to *fer-6(hc6)*, *fer-1(hc24)*, and *spe-9(hc88)* mutants to a similar extent as wild-type animals (Figs. 5C-E, Suppl. Table 1), suggesting that the presence of sperm and/or sperm precursors are sufficient to restore FUdR-dependence. Given that sperm or sperm precursors trigger meiotic maturation of oocytes and ovulation [28], we tested if these processes were important for FUdR-dependence. We mated *fem-3(e2006)* females (raised at the restrictive temperature of 25°C) to wild-type males, which restored ovulation and produced viable progeny (data not shown). We found that FUdR-treatment significantly enhanced mated *fem-3(e2006)* thermotolerance (Fig. 5F, Suppl. Table 1), showing that restoration of oogenesis and oocytic maturation sensitizes *fem-3(e2006)* animals to FUdR. Thus, these findings suggest that FUdR can confer stress resistance by inhibiting oocytic maturation.

### Loss of FEM-3 improves aspects of protein homeostasis

**Figure 6:**
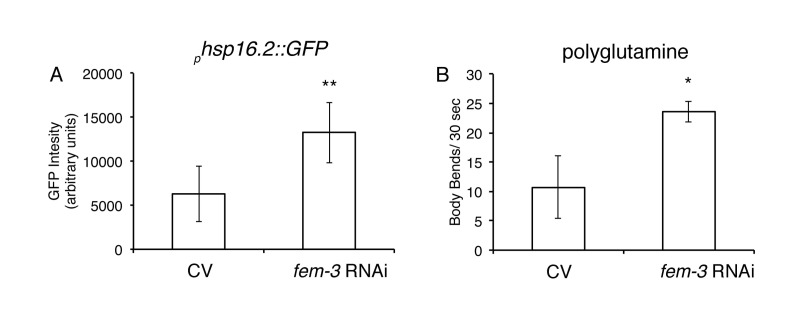
Loss of FEM-3 is sufficient to improve proteostasis **A**, Loss of *fem-3* via RNAi in *phsp16.2::GFP* animals significantly increases GFP expression compared to control vector (CV) animals after 1-hour heat shock at 35°C. (**=p<0.0001, Student's t-test). **B**, Loss of *fem-3* via RNAi in AM141 animals (Q40) significantly improves motility at day 5 of adulthood compared to CV-treated animals. (*p=.017, Student's t-test).

Next, we examined if loss of a FEM pathway was sufficient to improve protein homeostasis. While *fem-1(hc17)* and *fem-3(e2006)* mutants did not exhibit any enhanced thermotolerance compared to wild-type animals (Figs. 5A,B), we did observe that loss of FEM-3 expression via RNA interference (RNAi) significantly upregulated global expression of the transcriptional reporter *_p_hsp-16.2::GFP* after a 1-hour heat shock at 35°C compared to control animals (Fig. 6A). In addition, loss of FEM-3 via RNAi significantly improved motility in animals expressing expanded polyglutamine proteins in their muscle (AM141) that had been aged up to day 5 of adulthood (Fig. 6B). Thus, loss of the male sex-determining pathway FEM-3 is sufficient to improve aspects of protein homeostasis in *C. elegans*.

### FUdR improves healthspan in wild-type animals under proteotoxic conditions

**Figure 7:**
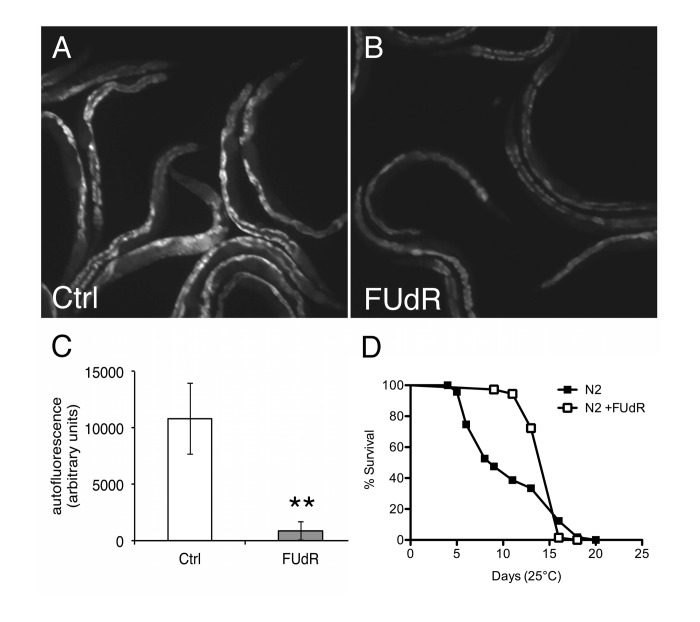
FUdR improves healthspan in wild-type animals at 25°C **A**, **B**, Photomicrographs of autofluorescence from day 9 wild-type N2 animals aged at 25°C in the absence or presence of FUdR (100 μg/ml). **C**, Quantification of **A**, **B**. FUdR-treatment significantly decreases the levels of autofluorescent age-pigments compared to untreated control animals. (**=p<0.0001, Student's t-test). **D**, FUdR-treatment significantly extends mean lifespan of wild-type N2 animals at 25°C (42.5%±17.6%).

Given that improved protein homeostasis is associated with health and longevity [15], we sought to re-examine the effects of FUdR on lifespan. Previous studies show no differences in longevity in animals maintained in the presence of FUdR at 20°C on live OP50 *E. coli* bacteria [6]. Similarly, we generally observed no significant differences in longevity at 20°C in the presence of FUdR (100 μg/ml) compared to untreated animals (Suppl. Table 2). However, at 25°C, we observed that FUdR-treatment potently inhibited the accumulation of autofluorescent molecules, or age pigments, a biomarker for health (Figs. 7A-C) [12]. Consistently, FUdR also significantly extended the mean lifespan of animals up to ~ 40% at 25°C, although no differences were observed in the maximal lifespan (Fig. 7D, Suppl. Table 2). The discrepancy between lifespan extension by FUdR at 20°C versus 25°C suggests that FUdR improves healthspan only under mildly proteotoxic conditions (25°C).

To determine if the mean lifespan extension observed at 25°C in the presence of FUdR was also dependent on FEM pathways, we performed lifespan analyses on *fem-3(e2006)* mutants. We observed no statistical differences in mean or maximal lifespan of *fem-3(e2006)* mutants in the absence or presence of FUdR (Suppl. Table 2). In contrast, FUdR-treatment significantly extended *daf-16(mu86)* lifespan (Suppl. Table 2). These findings are consistent with our thermotolerance data suggesting that FUdR requires the FEM pathways to confer protection and that this is independent of previously described germline signaling pathways.

## DISCUSSION

In this study, we report that administration of the small molecule DNA synthesis inhibitor, FUdR, confers protection under various conditions of proteotoxic stress. FUdR actively inhibits germline proliferation in the adult animal, suggesting that FUdR may interfere with a cell non-autonomous fertility signal to somatic tissues that impacts protein homeostasis. However, we have not ruled out that FUdR may also affect mitochondrial DNA replication or have non-specific, off-target effects. FUdR vastly improved pathologies in muscle-specific models of protein misfolding (perlecan, A-β, and polyglutamine). Previous studies have shown that polyglutamine misfolding in muscle tissues can be modulated via cell non-autonomous neuronal signals [31]. Further, recent studies show that inhibition of germline stem cells (GSCs) using a genetic mutant can improve protein turnover and muscle-specific models of protein misfolding via previously described germline signaling pathways, such as *daf-16, daf-12*, and *nhr-80* [4, 5]. However, we find that FUdR-induced stress resistance does not require any of these previously described germline signaling pathways. Thus, our findings suggest that FUdR may act via fertility signals that are distinct from GSCs. Consistently, we have identified that FUdR is dependent on oogenesis and oocytic maturation. Feminized animals with arrested oocytes are refractory to FUdR. The addition of sperm to feminized animals via mating is sufficient to trigger oocytic maturation and resume oogenesis, which restores FUdR-dependence. Consistently, mutants that are deficient in spermatogenesis but not oogenesis also respond to FUdR. Interestingly, Greer et al. also described signals from mature oocytes, independent of *daf-16* and *daf-12*, that are required to extend the lifespan of animals that have reduced expression of the methyl transferase, *ash-2* [32]. FUdR-treatment does not further extend the long lifespan of *ash-2* RNAi-treated animals while it does in control animals [32], suggesting that the modes of action for FUdR and loss of *ash-2* may overlap.

In addition to protecting in models of protein misfolding, we observed that FUdR caused global increased expression of the _p_*hsp-16.2::GFP* transcriptional reporter, primarily in the intestine. Overexpression of heat shock chaperones is sufficient to extend lifespan and can protect in models of Aβ and polyglutamine toxicity [33-35]. Thus, one possible mechanism for our observations is that that FUdR directly upregulates heat shock proteins (hsps). HSF-1 is the main transcription factor that modulates hsp expression and has also been implicated as a GSC signal [36]. Surprisingly, we find that the *hsf-1(sy441)* mutant, which exhibits severe impairment in the activation of *hsp16.2*, is still protected by FUdR under thermal stress, which suggests either that activation of *hsp16.2* is not the sole mechanism of FUdR and/or that *hsp16.2* transcription may be activated by pathways other than HSF-1.

In addition to chemical inhibition of fertility, we found that loss of FEM-3 is sufficient to increase the _p_*hsp-16.2::GFP* transcriptional reporter after heat stress and improve motility in a polyglutamine model of protein misfolding, but does not increase thermotolerance or lifespan, suggesting that inhibition of oocytic maturation can improve some, but not all, aspects of protein homeostasis. Previous studies have shown that sterile mutants have enhanced resistance to pathogen toxicity and are protected under conditions of low oxygen [7, 8], which is regulated by *daf-16*-dependent and -independent manners, respectively. In *Drosophila*, females that have mated or have increased egg production are susceptible to oxidative stress [37-39] suggesting that fertility can increase vulnerability to certain stressors. Our results point to the utility of small molecule screens to identify novel aspects of stress and aging biology. Here, we propose a novel regulatory mechanism whereby particular forms of sterility promote stress resistance and suppress the age-related pathology of protein misfolding. Based on fertility status, resources may be reallocated to promote protein homeostasis. This would be predicted to have effects on aging phenotypes consistent with the disposable soma theory.

## METHODS

### C. elegans strains used in this study

The following nematode strains were obtained from the *Caenorhabditis* Genetics Center (CGC, University of Minnesota) and cultured using standard conditions [40]. Bristol N2 (wild-type), CB4037 [*glp-1(e2141)*], AA86 [*daf-12(rh61rh411)*], CF1038 [*daf-16(mu86)*], BX165 [*nhr-80(tm1011)*], PS3551 [*hsf-1(sy441)*], BA6 [*fer-6(hc6)*], BA24 [*fer-1(hc24)*], BA671 [*spe-9(hc88)*], BA17[*fem-1(hc17)*], CB3844 [*fem-3(e2006)*]. For paralysis and motility experiments, the following strains were used: HE250 [*unc-52(e669su250)II*] [16], CL4176 [*smg-1 (cc546ts);dvIs27(myo-3::Aβ3-42 let 39UTR(pAF29))*]; *pRF4*] [17], AM141 [*rmIs133(P(unc-54) Q40::YFP)*] [18]. For reporter GFP experiments, CL2070 [*dvIs70(phsp16::GFP;pRF4)*] was used [20].

### FUdR preparation

Stock solutions of FUdR were prepared in deionized/distilled water at a concentration of 20 mg/ml. FUdR was diluted in deionized/distilled water and spotted onto NGM/OP50 *E. coli* plates to a final concentration of 100 μg/ml unless otherwise stated. Plates were dried at room temperature for 24 hours and subsequently stored at 4°C for no longer than 2 weeks.

### Lifespan assay and aging populations

Synchronized populations of *C. elegans* were cultured at the indicated temperatures (20°C or 25°C). Animals were transferred to FUdR-containing plates 48 hours after egg-lays and were maintained on FUdR plates for the remainder of the lifespan. Both water-treated controls and FUdR-treated populations were transferred daily to fresh plates for the first 5-6 days of adulthood (the reproductive period) and as necessary thereafter, typically every second or third day. Animals that crawled off the plates or died due to internal larval hatching (bagging) were censored from the population. Log rank (Mantel-Cox) statistics were used for analysis of lifespans using Prism™.

### Thermotolerance

Thermotolerance assays were performed as previously described [41]. Briefly, animals were reared from eggs at either 20°C or at the restrictive temperature of 25°C for temperature-sensitive mutants, and transferred to FUdR plates 48 hours later. After approximately 10-12 hours, the temperature was raised to 35°C and animals were scored for survival beginning after 6 hours and every hour thereafter until all animals were dead. Mated populations were generated by mating young hermaphrodites with young males (1:6 ratio of hermaphrodites to males). 24 hours later, mated hermaphrodites were removed from male populations and used as above. Log rank (Mantel-Cox) statistics were used to analyze thermotolerance results using Prism™.

### Microscopy and quantification

Animals used for microscopy were paralyzed in 1mM levamisole and mounted on agar pads with glass coverslips and immediately analyzed using an Olympus BX51 upright microscope. Similar results were obtained in the absence of levamisole (data not shown). Animals expressing GFP were analyzed using 470/40 nm excitation and 525/50 nm emission wavelengths. Autofluorescent age-pigment accumulation was analyzed using 350/50 nm excitation and 460/50 nm emission wavelengths. Approximately 20 animals per condition were used and normalized to the mid-line length of the animal. All experiments were repeated at least three times with similar results. Quantification of pixel densities was analyzed with Image J™.

### RNA interference

RNA interference (RNAi) was delivered to worms as previously described [42]. To achieve 100% sterility from *fem-3* RNAi (C01F6.4, Open Biosystems ORF RNAi library), animals were grown on RNAi bacteria for 2-3 consecutive generations before performing experiments.

### Paralysis and motility assays

Synchronous populations were reared at 15°C until the young adult/L4 stage (approximately 4-5 days from egg-lay); animals were then transferred to plates containing FUdR and simultaneously shifted to the restrictive temperature of 25°C. Paralysis was scored beginning after 24 hours for HE250 [*unc-52(e669su250)II*] strains and after 5 days for CL4176 [*smg-1 (cc546ts);dvIs27(myo-3::Ab3-42 let 39UTR(pAF29))*] strains. For motility assays with AM141 [*rmIs133(P(unc-54) Q40::YFP)*], day 5 adult animals were individually placed in a drop of M9 buffer and allowed to recover for 30 seconds. The number of body bends was then counted for 30 seconds.

## SUPPLEMENTARY TABLES AND FIGURES





## References

[R1] Kirkwood TB, Holliday R (1979). The evolution of ageing and longevity. Proc R Soc Lond B Biol Sci.

[R2] Hsin H, Kenyon C (1999). Signals from the reproductive system regulate the lifespan of C. elegans. Nature.

[R3] Arantes-Oliveira N, Apfeld J, Dillin A, Kenyon C (2002). Regulation of life-span by germ-line stem cells in Caenorhabditis elegans. Science New York, NY.

[R4] Vilchez D, Morantte I, Liu Z, Douglas PM, Merkwirth C, Rodrigues AP, Manning G, Dillin A (2012). RPN-6 determines C. elegans longevity under proteotoxic stress conditions. Nature.

[R5] Shemesh N, Shai N, Ben-Zvi A (2013). Germline stem cell arrest inhibits the collapse of somatic proteostasis early in Caenorhabditis elegans adulthood. Aging cell.

[R6] Van Raamsdonk JM, Hekimi S (2011). FUdR causes a twofold increase in the lifespan of the mitochondrial mutant gas-1. Mech Ageing Dev.

[R7] Miyata S, Begun J, Troemel ER, Ausubel FM (2008). DAF-16-dependent suppression of immunity during reproduction in Caenorhabditis elegans. Genetics.

[R8] Mendenhall AR, LeBlanc MG, Mohan DP, Padilla PA (2009). Reduction in ovulation or male sex phenotype increases long-term anoxia survival in a daf-16-independent manner in Caenorhabditis elegans. Physiol Genomics.

[R9] Aitlhadj L, Sturzenbaum SR (2010). The use of FUdR can cause prolonged longevity in mutant nematodes. Mech Ageing Dev.

[R10] Davies SK, Leroi AM, Bundy JG (2012). Fluorodeoxyuridine affects the identification of metabolic responses to daf-2 status in Caenorhabditis elegans. Mech Ageing Dev.

[R11] Herndon LA, Schmeissner PJ, Dudaronek JM, Brown PA, Listner KM, Sakano Y, Paupard MC, Hall DH, Driscoll M (2002). Stochastic and genetic factors influence tissue-specific decline in ageing C. elegans. Nature.

[R12] Gerstbrein B, Stamatas G, Kollias N, Driscoll M (2005). In vivo spectrofluorimetry reveals endogenous biomarkers that report healthspan and dietary restriction in Caenorhabditis elegans. Aging cell.

[R13] David DC, Ollikainen N, Trinidad JC, Cary MP, Burlingame AL, Kenyon C (2010). Widespread protein aggregation as an inherent part of aging in C. elegans. PLoS biology.

[R14] Reis-Rodrigues P, Czerwieniec G, Peters TW, Evani US, Alavez S, Gaman EA, Vantipalli M, Mooney SD, Gibson BW, Lithgow GJ, Hughes RE (2012). Proteomic Analysis of Age-dependent Changes in Protein Solubility Identifies Genes that Modulate Lifespan. Aging cell.

[R15] Alavez S, Vantipalli MC, Zucker DJ, Klang IM, Lithgow GJ (2011). Amyloid-binding compounds maintain protein homeostasis during ageing and extend lifespan. Nature.

[R16] Mackenzie JM, Garcea RL, Zengel JM, Epstein HF (1978). Muscle development in Caenorhabditis elegans: mutants exhibiting retarded sarcomere construction. Cell.

[R17] Drake J, Link CD, Butterfield DA (2003). Oxidative stress precedes fibrillar deposition of Alzheimer's disease amyloid beta-peptide (1-42) in a transgenic Caenorhabditis elegans model. Neurobiology of aging.

[R18] Morley JF, Brignull HR, Weyers JJ, Morimoto RI (2002). The threshold for polyglutamine-expansion protein aggregation and cellular toxicity is dynamic and influenced by aging in Caenorhabditis elegans. Proceedings of the National Academy of Sciences of the United States of America.

[R19] Walker GA, Lithgow GJ (2003). Lifespan extension in C. elegans by a molecular chaperone dependent upon insulin-like signals. Aging cell.

[R20] Link CD, Cypser JR, Johnson CJ, Johnson TE (1999). Direct observation of stress response in Caenorhabditis elegans using a reporter transgene. Cell stress & chaperones.

[R21] Austin J, Kimble J (1987). glp-1 is required in the germ line for regulation of the decision between mitosis and meiosis in C. elegans. Cell.

[R22] Kodoyianni V, Maine EM, Kimble J (1992). Molecular basis of loss-of-function mutations in the glp-1 gene of Caenorhabditis elegans. Mol Biol Cell.

[R23] McColl G, Rogers AN, Alavez S, Hubbard AE, Melov S, Link CD, Bush AI, Kapahi P, Lithgow GJ (2010). Insulin-like signaling determines survival during stress via posttranscriptional mechanisms in C. elegans. Cell Metab.

[R24] Goudeau J, Bellemin S, Toselli-Mollereau E, Shamalnasab M, Chen Y, Aguilaniu H (2011). Fatty acid desaturation links germ cell loss to longevity through NHR-80/HNF4 in C. elegans. PLoS biology.

[R25] Hajdu-Cronin YM, Chen WJ, Sternberg PW (2004). The L-type cyclin CYL-1 and the heat-shock-factor HSF-1 are required for heat-shock-induced protein expression in Caenorhabditis elegans. Genetics.

[R26] Doniach T, Hodgkin J (1984). A sex-determining gene, fem-1, required for both male and hermaphrodite development in Caenorhabditis elegans. Dev Biol.

[R27] Hodgkin J (1986). Sex determination in the nematode C. elegans: analysis of tra-3 suppressors and characterization of fem genes. Genetics.

[R28] McCarter J, Bartlett B, Dang T, Schedl T (1999). On the control of oocyte meiotic maturation and ovulation in Caenorhabditis elegans. Dev Biol.

[R29] Ward S, Argon Y, Nelson GA (1981). Sperm morphogenesis in wild-type and fertilization-defective mutants of Caenorhabditis elegans. The Journal of cell biology.

[R30] Singson A, Mercer KB, L'Hernault SW (1998). The C. elegans spe-9 gene encodes a sperm transmembrane protein that contains EGF-like repeats and is required for fertilization. Cell.

[R31] Prahlad V, Morimoto RI (2011). Neuronal circuitry regulates the response of Caenorhabditis elegans to misfolded proteins. Proceedings of the National Academy of Sciences of the United States of America.

[R32] Greer EL, Maures TJ, Hauswirth AG, Green EM, Leeman DS, Maro GS, Han S, Banko MR, Gozani O, Brunet A (2010). Members of the H3K4 trimethylation complex regulate lifespan in a germline-dependent manner in C. elegans. Nature.

[R33] Hsu AL, Murphy CT, Kenyon C (2003). Regulation of aging and age-related disease by DAF-16 and heat-shock factor. Science.

[R34] Fonte V, Kipp DR, Yerg J, Merin D, Forrestal M, Wagner E, Roberts CM, Link CD (2008). Suppression of in vivo beta-amyloid peptide toxicity by overexpression of the HSP-16.2 small chaperone protein. The Journal of biological chemistry.

[R35] Morley JF, Morimoto RI (2004). Regulation of longevity in Caenorhabditis elegans by heat shock factor and molecular chaperones. Mol Biol Cell.

[R36] Hansen M, Hsu AL, Dillin A, Kenyon C (2005). New genes tied to endocrine, metabolic, and dietary regulation of lifespan from a Caenorhabditis elegans genomic RNAi screen. PLoS genetics.

[R37] Rush B, Sandver S, Bruer J, Roche R, Wells M, Giebultowicz J (2007). Mating increases starvation resistance and decreases oxidative stress resistance in Drosophila melanogaster females. Aging cell.

[R38] Salmon AB, Marx DB, Harshman LG (2001). A cost of reproduction in Drosophila melanogaster: stress susceptibility. Evolution.

[R39] Wang Y, Salmon AB, Harshman LG (2001). A cost of reproduction: oxidative stress susceptibility is associated with increased egg production in Drosophila melanogaster. Exp Gerontol.

[R40] Sulston JHJ, WB W (1988). Methods. The Nematode Caenorhabditis elegans.

[R41] Lithgow GJ, White TM, Melov S, Johnson TE (1995). Thermotolerance and extended life-span conferred by single-gene mutations and induced by thermal stress. Proceedings of the National Academy of Sciences of the United States of America.

[R42] Kamath RS, Martinez-Campos M, Zipperlen P, Fraser AG, Ahringer J (2001). Effectiveness of specific RNA-mediated interference through ingested double-stranded RNA in Caenorhabditis elegans. Genome Biol.

