# Supplementary material for: A DNA synthesis inhibitor is protective against proteotoxic stressors via modulation of fertility pathways in Caenorhabditis elegans
Source: Aging (Albany NY). 2013 Oct 10;5(10):759–69. doi: 10.18632/aging.100605 (PMC3838778; doi:10.18632/aging.100605)
Supplement: Supplementary file 1 [file aging-05-759-s001.pdf]

## SUPPLEMENTARY FIGURES

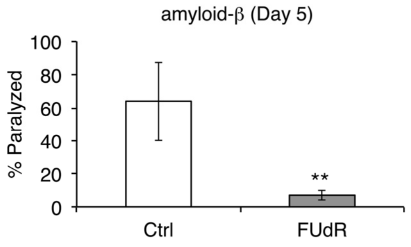


**Suppl. Figure 1. Transient administration of FUdR during spermatogenesis rescues Aβ pathology**. Temperature-sensitive strain, CL4176 [*smg-1 (cc546ts); dvIs27(myo-3::Ab3–42 let 39UTR(pAF29))*], which expresses amyloid-$\beta_{3\text{-}42}$ peptide, was scored for paralysis on day 5 of adulthood after transient administration of FUdR (100 μg/ml) during spermatogenesis. FUdR-treatment significantly reduces paralysis compared to control animals. (**=p<0.01, Student's t-test).

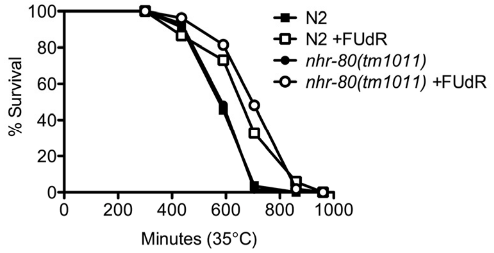


**Suppl. Figure 2. FUdR confers thermotolerance via germline signals that are independent of *nhr-80.*** FUdR-treatment (100 μg/ml) significantly extends *nhr-80(tm1011)* survival to a similar extent as wild-type N2 animals. Animals were reared at 25°C and shifted to 35°C as young adults.

## SUPPLEMENTARY TABLES

Please browse full text version of this manuscript to see the the Supplementary **Table S1** and **Table S2.**
