# Supplementary material for: A DNA synthesis inhibitor is protective against proteotoxic stressors via modulation of fertility pathways in Caenorhabditis elegans
Source: Aging (Albany NY). 2013 Oct 10;5(10):759–69. doi: 10.18632/aging.100605 (PMC3838778; doi:10.18632/aging.100605)
Supplement: Supplementary file 2 [file aging-05-759-s002.docx]

**Supplementary Table 1: Thermotolerance Assays (35 °C)**

| Strain/ Treatment* | Mean Survival (minutes) | n | Log- rank p-value (compared to control ) |
|---|---|---|---|
| **Experiment #1** | | | |
| N2 control | 619 | 56 | |
| N2 +FUdR | 713 | 47 | <0.0001 |
| *glp-1(e2141)* control | 790 | 50 | |
| *glp-1(e2141)* +FUdR | 726 | 54 | 0.0206 |
| **Experiment #2** | | | |
| N2 control | 569 | 51 | |
| N2 +FUdR | 828 | 29 | <0.0001 |
| *glp-1(e2141)* control | 761 | 40 | |
| *glp-1(e2141)* +FUdR | 684 | 47 | 0.1242 |
| **Experiment #3** | | | |
| N2 control | 645 | 40 | |
| N2 +FUdR | 741 | 28 | 0.0049 |
| *glp-1(e2141)* control | 871 | 37 | |
| *glp-1(e2141)* +FUdR | 808 | 39 | 0.1421 |
| **Experiment #1** | | | |
| N2 control | 525 | 56 | |
| N2 +FUdR | 803 | 39 | <0.0001 |
| *daf-12((rh61rh411)* control | 519 | 56 | |
| *daf-12((rh61rh411)* +FUdR | 789 | 44 | <0.0001 |
| *daf-16(mu86)* control | 455 | 59 | |
| *daf-16(mu86)* +FUdR | 738 | 58 | <0.0001 |
| **Experiment #2** | | | |
| N2 control | 499 | 54 | |
| N2 +FUdR | 770 | 46 | <0.0001 |
| *daf-12(rh61rh411)* control | 514 | 54 | |
| *daf-12(rh61rh411)*+FUdR | 739 | 48 | <0.0001 |
| *daf-16(mu86)* control | 488 | 55 | |
| *daf-16(mu86)* +FUdR | 757 | 56 | <0.0001 |
| **Experiment #1** | | | |
| N2 control | 634 | 57 | |
| N2 +FUdR | 710 | 52 | <0.0001 |
| *nhr-80(tm1011)* | 638 | 58 | |
| *nhr-80(tm1011)* | 755 | 54 | <0.0001 |

| Strain/ Treatment* | Mean Survival (minutes) | n | Log- rank p-value (compared to control ) |
|---|---|---|---|
| **Experiment #1** | | | |
| N2 control | 569 | 51 | |
| N2 +FUdR | 828 | 29 | <0.0001 |
| *hsf-1(sy441)* control | 588 | 43 | |
| *hsf-1(sy441)* +FUdR | 790 | 41 | <0.0001 |
| **Experiment #2** | | | |
| *hsf-1(sy441)* control | 553 | 49 | |
| *hsf-1(sy441)* +FUdR | 750 | 48 | <0.0001 |
| **Experiment #1** | | | |
| N2 control | 714 | 49 | |
| N2 +FUdR | 842 | 51 | <0.0001 |
| *fer-6(hc6)* control | 645 | 50 | |
| *fer-6(hc6)* +FUdR | 807 | 51 | <0.0001 |
| **Experiment #1** | | | |
| N2 control | 645 | 53 | |
| N2 +FUdR | 761 | 53 | <0.0001 |
| *fer-1(hc24)* control | 664 | 49 | |
| *fer-1(hc24)* +FUdR | 733 | 53 | <0.0001 |
| **Experiment #2** | | | |
| N2 control | 582 | 50 | |
| N2 +FUdR | 758 | 42 | <.0.0001 |
| *fer-1(hc24)* control | 656 | 50 | |
| *fer-1(hc24)* +FUdR | 707 | 40 | 0.0014 |
| **Experiment #1** | | | |
| N2 control | 699 | 52 | |
| N2 +FUdR | 803 | 46 | <0.0001 |
| *spe-9(hc88)* control | 652 | 52 | |
| *spe-9(hc88)* +FUdR | 782 | 50 | <0.0001 |
| **Experiment #2** | | | |
| N2 control | 648 | 48 | |
| N2 +FUdR | 758 | 52 | <0.0001 |
| *spe-9(hc88)* control | 644 | 54 | |
| *spe-9(hc88)* +FUdR | 749 | 48 | <0.0001 |

| Strain/ Treatment* | Mean Survival (minutes) | n | Log- rank p-value (compared to control ) |
|---|---|---|---|
| **Experiment #1** | | | |
| N2 control | 698 | 49 | |
| N2 +FUdR | 777 | 46 | <0.0001 |
| *fem-1(hc17)* control | 725 | 34 | |
| *fem-1(hc17)* +FUdR | 680 | 38 | 0.0864 |
| **Experiment #2** | | | |
| N2 control | 700 | 49 | |
| N2 +FUdR | 859 | 52 | <0.0001 |
| *fem-1(hc17)* control | 703 | 49 | |
| *fem-1(hc17)* +FUdR | 704 | 47 | 0.6488 |
| **Experiment #1** | | | |
| N2 control | 665 | 52 | |
| N2 +FUdR | 755 | 53 | <0.0001 |
| *fem-3(e2006)* control | 670 | 50 | |
| *fem-3(e2006)* +FUdR | 650 | 49 | 0.1314 |
| **Experiment #2** | | | |
| N2 control | 631 | 54 | |
| N2 +FUdR | 699 | 53 | 0.0002 |
| *fem-3(e2006)* control | 667 | 40 | |
| *fem-3(e2006)* +FUdR | 640 | 42 | 0.1754 |
| **Experiment #3** | | | |
| N2 control | 747 | 55 | |
| N2 +FUdR | 891 | 52 | <0.0001 |
| *fem-3(e2006)* control | 801 | 60 | |
| *fem-3(e2006)* +FUdR | 838 | 47 | 0.6773 |
| **Experiment #1** | | | |
| *fem-3(e2006)* mated | 671 | 26 | |
| *fem-3(e2006)* mated +FUdR | 731 | 29 | 0.0389 |
| **Experiment #2** | | | |
| *fem-3(e2006)* mated | 581 | 29 | |
| *fem-3(e2006)* mated +FUdR | 681 | 31 | 0.0004 |

## Supplementary Table 2: Lifespan Assays

| Temperature | Strain/Treatment* | Mean Survival (days) | n (censored) | Log- rank p-value (compared to control strain) |
|---|---|---|---|---|
| **20 °C** | **Experiment #1** | | | |
| | N2 control | 17 | 51(25) | |
| | N2 +FUdR | 17 | 75(5) | 0.2091 |
| **20 °C** | **Experiment #2** | | | |
| | N2 control | 15 | 62 (12) | |
| | N2 +FUdR | 15 | 33 (37) | 0.9273 |
| **20 °C** | **Experiment #3** | | | |
| | N2 control | 14 | 46 (29) | |
| | N2 +FUdR | 16 | 70 (3) | 0.0189 |
| **25 °C** | **Experiment #1** | | | |
| | N2 control | 9 | 34 (24) | |
| | N2 +FUdR | 13 | 67 (0) | <0.0001 |
| **25 °C** | **Experiment #2** | | | |
| | N2 control | 12 | 39(28) | |
| | N2 +FUdR | 15 | 76(3) | 0.0004 |
| **25 °C** | **Experiment #3** | | | |
| | N2 control | 8 | 71 (4) | |
| | N2 +FUdR | 13 | 60 (4) | <0.0001 |
| **25 °C** | **Experiment #1** | | | |
| | N2 control | 10 | 36(15) | |
| | N2 +FUdR | 13 | 48(0) | 0.001 |
| | *fem-3(e2006)* control | 11 | 48(1) | |
| | *fem-3(e2006)* +FUdR | 12 | 47(4) | 0.3961 |
| **25 °C** | **Experiment #2** | | | |
| | N2 control | 10 | 37(22) | |
| | N2 +FUdR | 12 | 53(1) | 0.0091 |
| | *fem-3(e2006)* control | 12 | 53(1) | |
| | *fem-3(e2006)* +FUdR | 14 | 56(0) | 0.161 |

| | | | | |
|---|---|---|---|---|
| **25 °C** | **Experiment #1** | | | |
| | N2 control | 11 | 59(17) | |
| | N2 +FUdR | 15 | 70(3) | <0.0001 |
| | *daf-16(mu86)* control | 9 | 55(16) | |
| | *daf-16(mu86)* +FUdR | 11 | 85(1) | 0.0004 |
| **25 °C** | **Experiment #2** | | | |
| | N2 control | 10 | 47(16) | |
| | N2 +FUdR | 13 | 68(0) | 0.0036 |
| | *daf-16(mu86)* control | 8 | 37(23) | |
| | *daf-16(mu86)* +FUdR | 10 | 72(0) | <0.0001 |
